# The effect of different criteria on the number of patients blind from open-angle glaucoma

**DOI:** 10.1186/1471-2415-11-31

**Published:** 2011-11-10

**Authors:** Anders Heijl, Johan Aspberg, Boel Bengtsson

**Affiliations:** 1 Department of Clinical Sciences in Malmö, Ophthalmology, Lund University, Malmö, Sweden

## Abstract

**Background:**

The prevalence of blindness and visual impairment from glaucoma is influenced by the criteria used to define these entities, which differ between countries and regions, as well as among published reports. The objective of the present study was to ascertain the extent to which different criteria of blindness and visual impairment influence estimates of the number of patients classified as blind or visually impaired by glaucoma in a clinic-based population.

**Methods:**

We conducted a retrospective chart review of 914 patients with open-angle glaucoma to compare numbers of patients identified as visually impaired with and without considering visual field status. We also compared proportions classified using World Health Organisation (WHO) and United States (US) blindness criteria, and applying a new US Social Security Administration (SSA) disability criterion: perimetric mean deviation (MD) ≤ -22 dB.

**Results:**

Forty patients (4.4%) were bilaterally blind from glaucoma by the WHO criteria. Fifty-two (5.7%) were blind by the the US criterion. Assessing only visual acuity, 14 (1.5%) patients were blind by the WHO criteria and 24 (2.6%) by the US definition. Eighty-five (9.3%) met the US SSA disability criterion. Among those, 52 were impaired also by the WHO definition. No patients impaired according to the WHO criteria had MD values better than -22 dB.

**Conclusions:**

Excluding visual field status will seriously underestimate the prevalence of glaucoma blindness. In our patient population, 30% more patients were classified as blind by the US than by the WHO definition. Also, 60% more were identified as visually impaired by the US SSA criterion than by the WHO criteria. Visual field assessment is vital to determine visual impairment caused by glaucoma.

## Background

Open-angle glaucoma is the second most common cause of blindness globally [1]. It is important that we have knowledge about the prevalence of visual impairment caused by glaucoma in order to enable correct allocation of resources for glaucoma care, and also to allow evaluation of the effects of treatment and potential benefits of population screening.

Evaluation of the prevalence of blindness and visual impairment from glaucoma is influenced by the criteria used to define these entities, which differ between countries and regions. The definition of visual impairment stipulated by the World Health Organisation (WHO) can be considered the current gold standard in this context. However, many countries use their own criteria for blindness. An example of this is the definition used in the United States here designated the US criteria, which is also widely accepted and used in other countries. The number of people identified as blind from glaucoma should be higher when using the US definition than with the WHO definition, since the latter includes stricter criteria for blindness both by visual acuity (VA) and visual field loss.

In some cases, the prevalence of visual impairment (blindness and low vision) in glaucoma is studied and reported based on VA alone, i.e., not including visual field status [2-21]. It seems obvious that this approach will yield falsely low estimates of the prevalence, because visual impairment will not be recognised in patients who have end-stage glaucomatous visual field loss with preserved central VA.

The US Social Security Administration (SSA) recently endorsed use of a new criterion for disability determinations, the perimetric mean deviation (MD). This ruling [22] states that an MD of -22 dB on a 30-2 Humphrey threshold visual field corresponds approximately to a constriction of the visual field to less than 20° of fixation, and recommends an MD of ≤ -22 dB as a visual field criterion to define disability.

The objective of the present study was to ascertain the extent to which different criteria of blindness and visual impairment influence estimates of the number of patients classified as blind or visually impaired by glaucoma. Our primary aim was to investigate how omitting visual field status from criteria affects the rate of visual impairment. We also wanted to compare the number of patients identified as blind from glaucoma by the US and the WHO glaucoma blindness definitions, respectively, and to estimate the number classified as disabled by the US SSA visual field criterion compared to the WHO criteria.

## Methods

### Patients

We performed a retrospective chart review of patients diagnosed with open-angle glaucoma who visited the Department of Ophthalmology at Malmö University Hospital between 1 June 2004 and 31 May 2006. The great majority of these patients had reproducible visual field defects defined as a Glaucoma Hemifield Test "outside normal limits" on a SITA Standard test performed on a Humphrey Field Analyzer (HFA; Carl Zeiss Meditec Inc., Dublin, CA, USA), compatible with glaucoma, and not explained by other ocular or neurological disorders.

We selected patients born on days 1-15 of each month and used a study period of 2 years so that we would not miss those who made only infrequent visits to the Malmö Department of Ophthalmology. Glaucoma patients not treated primarily at this department (e.g., those referred for cataract surgery, laser treatment, or a second opinion) were not eligible, because our records on such cases contained insufficient data for reliable classifications.

For each patient, we recorded VA, perimetric findings in both eyes (as MD values of SITA Standard 30-2 tests in the great majority of cases, but also by Goldmann kinetic perimetry in a few), age, and presence/absence of visual impairment determined by the various criteria studied. Patients who met the WHO blindness criterion for VA and lacked visual field data were assigned an MD value of -30 dB.

The study was approved by the Regional Ethics Board in Lund, Sweden, and the tenets of the Declaration of Helsinki were followed.

### Comparisons of criteria

The term visual impairment includes both low vision and blindness. Here, bilateral visual impairment was based on best-corrected VA and/or visual field status in the best eye, and hence a person with one blind eye and low vision in the other was considered to have low vision.

Each patient was evaluated for the presence of visual impairment using these different criteria:

1. Low vision and blindness according to the WHO criteria

2. Blindness according to the US criteria

3. Impairment according to the perimetric MD value criterion suggested by the US SSA [22] (Table 1)

**Table 1:** Visual impairment criteria*

	WHO	US	US SSA
Low vision	VA < 0.3 (20/70) and/or a constriction of the central visual field to < 20°	VA < 0.5 (20/40).	MD ≤ -22 dB in both eyes.
	
Blindness	VA < 0.05 (20/400) and/or a constriction of the central visual field to < 10°	VA ≤ 0.1 (6/60) and/or a constriction of the central visual field to < 20°	

*Visual impairment = low vision + blindness.

VA, visual acuity; MD, mean deviation; dB, decibel.

4. Low vision and blindness based on VA alone, thus omitting visual field data

Visual field status is not included in the US criterion, defining low vision as VA < 0.5. Therefore, we chose not to register patients classified as having low vision according to this criterion.

**Figure 1:**
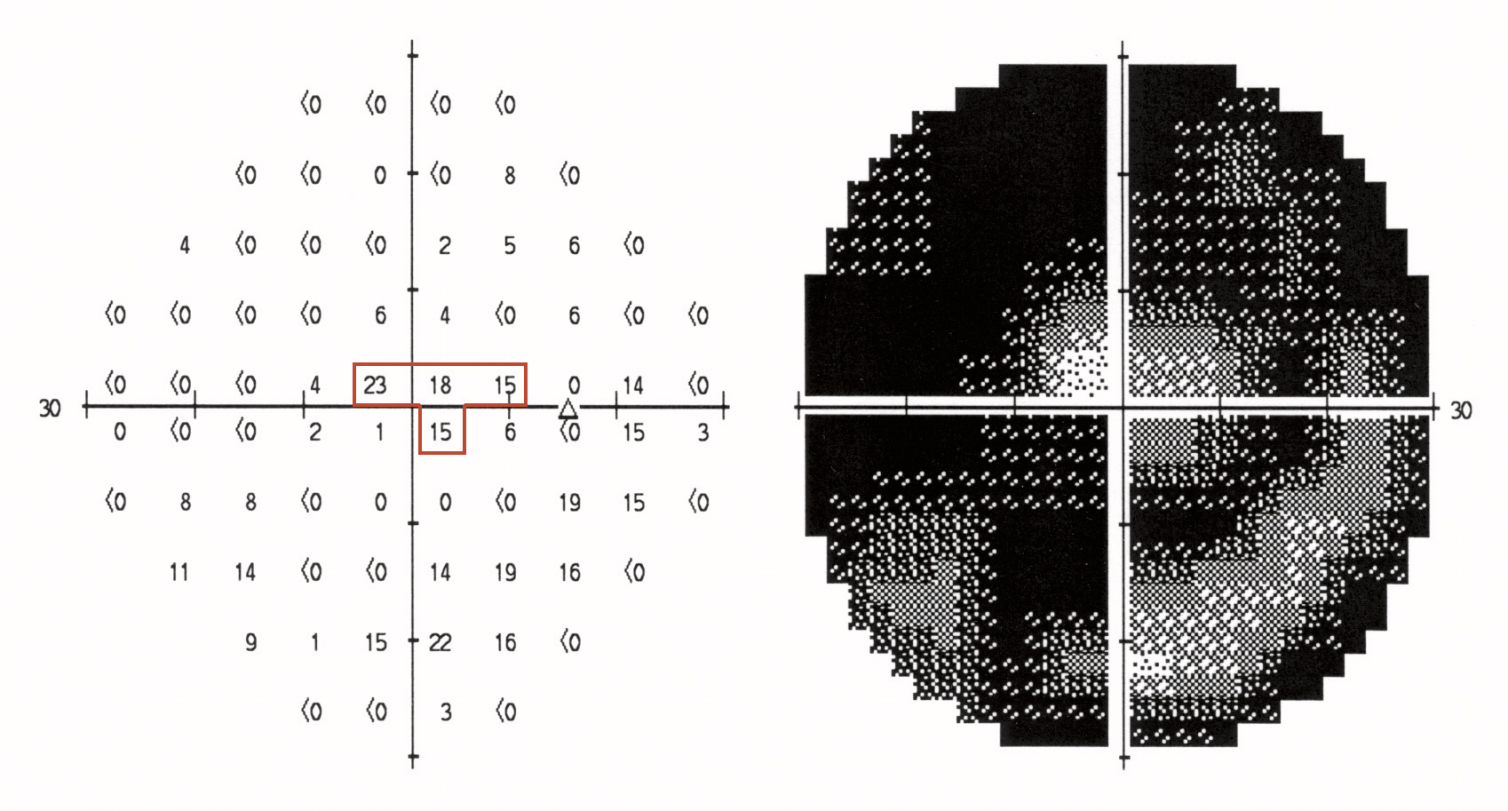
**Calculation of the diameter of the remaining visual field**. The central visual field is indicated by the pseudoisopter (red line). The distance is 6° between each test point location and 3° between test points and the pseudoisopter. The pseudoisopter is used to calculate the widest diameter of the remaining central visual field. This field is constricted to 18° around the point of fixation.

We calculated the diameter of the remaining visual field as recommended by the SSA [23]. Pseudoisopters were drawn on the HFA numerical threshold dB printouts, midway between test point locations with threshold sensitivity values of 10 dB or better, and points with sensitivity less than 10 dB (Figure 1).

The causes of visual loss were determined for all patients with visual impairment. For each impaired eye, the disease that initially contributed to visual loss was noted as the leading cause. However, if the time for impairment could not be established, the disease that was deemed to contribute most to the impairment was registered as the main cause. Visual field data were available in most cases in which glaucoma was deemed to be the main cause. Such data were lacking in some cases, such as for elderly patients with end-stage glaucoma who could not participate in visual field testing, or when VA was so low that visual field testing had been abandoned. Classification in those cases was based solely on VA.

We used both the WHO criteria and the US criterion (blindness only) to determine the number of bilaterally blind/visually impaired patients, and we calculated the effect of omitting visual field status from the definition of visual impairment. We also investigated the difference in the numbers of patients defined as blind by the WHO and US criteria. The numbers of patients classified as impaired with WHO criteria and the US SSA visual field criterion were compared.

### Statistical analyses

We applied descriptive statistics to compare the US and WHO criteria regarding the number of patients identified as blind and visually impaired, and the number of patients with and without using visual field data. The number classified as blind and visually impaired will always be larger when visual field criteria are applied than when they are omitted, because all patients who are blind according to VA criteria alone are also blind when field data are included. Similarly, all patients who are identified as blind according to the WHO criteria are also blind according to the less stricter US criteria. Analyses of numbers of blind according to WHO and US criteria and with and without taking visual fields into account were, therefore, purely descriptive, and no analyses for significances were performed.

## Results

A total of 914 eligible patients were evaluated. Their mean age was 79 years (range 30-100 years). Nearly all the patients were Caucasians.

**Figure 2:**
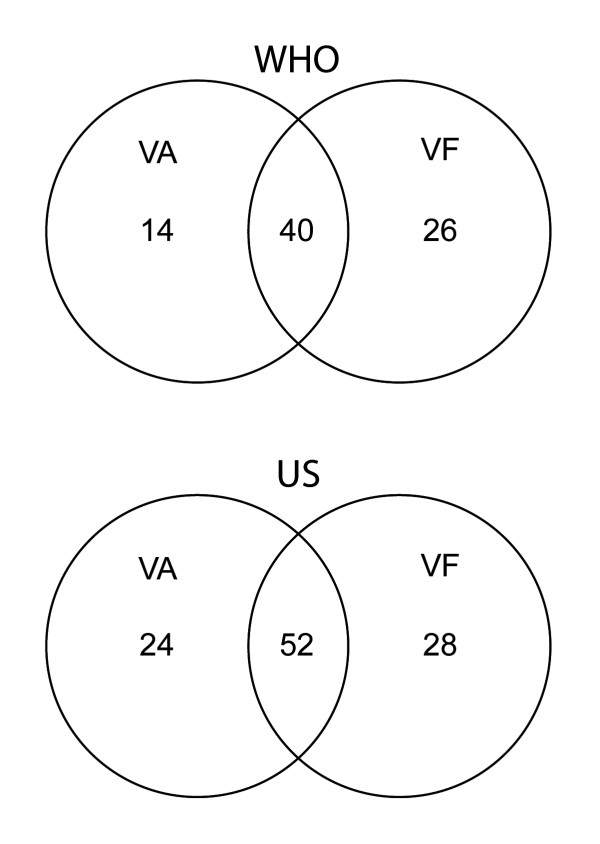
**Blindness by visual acuity or field**. Number of blind patients (best eye) using visual acuity (VA) or visual field (VF) data with WHO and US criteria.

Table 2 presents the total numbers of patients identified as blind or visually impaired by the WHO and US criteria with and without taking visual field status into account. The number of blind patients was 30% higher according to the US criteria than the WHO criteria. Using the WHO criteria, the number of patients classified as blind was 40 when visual field was included but only 14 when visual field status was omitted, which represents a 65% reduction (Figure 2). Similarly, the WHO criteria identified visual impairment in only 37 patients when visual field status was ignored as compared to 58 patients when field status was included, a 36% reduction. Using the US criterion, the number of blind patients was only 24 when visual fields were omitted compared to 52 when field status was included, a reduction of 54%.

**Table 2:** Influence of visual field status on the number of patients classified as blind/visually impaired from glaucoma

	Visual fields + VA* No. (% of all patients)	VA alone No. (%)
WHO - blindness	40 (4.4%)	14 (1.5%)

WHO - visual impairment	58 (6.3%)	37 (4.0%)

US - blindness	52 (5.7%)	24 (2.6%)

* VA, visual acuity.

Eighty-five patients (9.3%) met the SSA criterion of bilateral visual impairment caused by glaucoma in both eyes with measured or assigned MD values ≤ -22 dB. Among those, 52 were visually impaired also by the WHO definition. No patients who were impaired according to the WHO criteria had MD values better than -22 dB.

Fifty-four patients had visual fields tested in one eye only, and 36 had no field tests at all. In 54 (60%) of those cases, the reason tests were not performed was that the patients already had very low VA and totally cupped discs at the initial visit, and in 28 cases it was because the patients' visual impairment was due to a cause other than glaucoma. For nine patients, perimetric results were not available for the study period or had been obtained only by testing on the Competer perimeter. Three patients were considered unable to do undergo perimetric testing.

## Discussion

**Figure 3:**
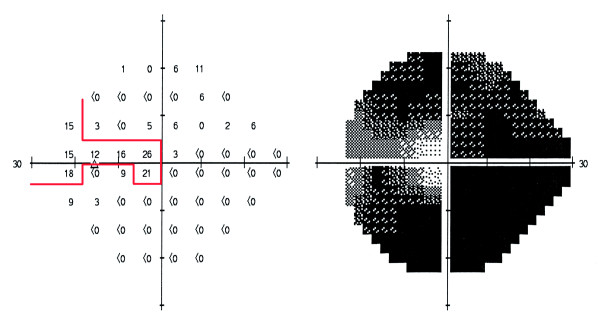
**Example of the classification of a visual field**. This field does not fulfill WHO or US blindness criteria, despite very advanced field loss (MD: -27.19).

The most important finding of the current study is that a very large proportion of the visually impaired glaucoma patients were not identified as being visually impaired when only VA criteria were used to define such low vision. This can be compared with an investigation performed by Hattenhauer et al [24], in which the risk of bilateral blindness was calculated for patients 20 years after a glaucoma diagnosis had been made using the US definition of blindness. Those authors found that the risk was 9% based on both visual field status and VA together but was 5% when based on VA alone, which is similar to our results. The visual field data in the study by Hattenhauer and colleagues came mainly from kinetic Goldmann perimetry. The Baltimore Eye Survey [25] using similar visual field testing, on the other hand suggested that the number of patients classified as having bilateral blindness would increase by at most 25% when combining visual field data and VA compared to if evaluation were based on VA alone. The visual field criteria for blindness and for visual impairment are quite restrictive. In glaucoma the remaining visual field is seldom circular, and many eyes are not classified as blind despite very small remaining field areas. A narrow segment of the field with test points with sensitivity values of 10 dB or better, that extends outside 10° from fixation may be enough for an eye not to fulfill any of the two blindness criteria (Figure 3). It is, therefore, obvious that impairment caused by glaucomatous field loss requires loss of a very considerable proportion of normal function and has large consequences if bilateral, and that field loss is a very important part of the visual burden caused by glaucoma.

Our results indicate that it is very likely that glaucoma impairment and blindness have been considerably underestimated in papers reporting rates of blindness determined using VA data, but omitting visual field status [3,4,15,18]. Furthermore, glaucoma must have been substantially underestimated as a cause of blindness in numerous population studies which have relied solely on VA [2,5-14,16,17,19-21]. Relying on visual acuity alone results in under-estimation of impairment caused by other disease, e.g., in onchocerciasis [26].

The number of patients classified as blind from glaucoma differs between investigations using the US criteria and those applying the WHO criteria. To our knowledge only two studies including visual field status has used both the WHO and the US definition to evaluate glaucoma blindness [27,28]. However, very few patients in the cited investigations were blind from glaucoma, and thus the results of that evaluation do not allow any conclusions to be drawn about the difference in the numbers of blind identified using these two approaches.

We found it interesting to study results obtained using the US SSA criteria for visual impairment. To our knowledge no other investigators have published visual impairment data based on MD values. It would no doubt be easier to use this MD-based criterion in surveys comparing visual impairment than performing actual measurements of visual field constriction, and that would also facilitate comparison of observations made in different studies.

This study had several strengths. Automated HFA perimetry is standard practice in our clinical setting, and other types of perimetry are rarely used. Ophthalmic practice in Sweden is somewhat unusual in that glaucoma care is delivered predominantly by the public health services. Our hospital provides primary glaucoma care for approximately three quarters of all patients with this disease in the catchment area. The current study was cross-sectional in design and included a rather large number of visually impaired patients. Therefore, our results should be reasonably representative of clinic-based populations. One may speculate on whether the proportions of individuals classified as blind from glaucoma using visual acuity alone or taking both visual acuity and fields into account are the same in clinic-based cohorts and in the population. If so the true prevalences of glaucoma blindness could be more than twice as large as those reported from many countries, and a "correction/multiplication factor" of 2.2 (US) or 2.9 (WHO) could be used to help provide at least a rough estimate of the total number of blind glaucoma patients in areas where such blindness has been estimated using visual acuity alone. Similar multiplication factors have been used by WHO to estimate low vision in areas where only blindness and not low vision have been assessed [29]. Reduced visual acuity seems to influence measurable reduction of quality of life earlier than field loss, and measurable utility may not be affected until end stage visual field status in the better eye [30,31]. It is, therefore, even possible that the proportion of undiagnosed individuals with glaucoma in the population who are blind according to visual field criteria, but not because of poor visual acuity, are even more common in the population than in clinic-based cohorts.

A relative weakness of our investigation is that it was retrospective, and therefore some data were incomplete or missing. However, missing data constitute a problem in prospective studies as well. Visual field testing can also pose a problem in very old patients or in elderly with very low vision, who might have to be followed without such testing during their last few years of life. Also, considering prospective studies, some institutionalised patients are lost to follow-up, and some continue to come for examinations but no subjective measurements of visual function can be performed. The great majority of our patients were subjected to regular follow-up that included visual field testing, and there was little occurrence of incomplete or missing data.

## Conclusions

In conclusion, we found that the number of patients with bilateral blindness from glaucoma was greatly underestimated when the evaluation was based solely on VA testing. The number of bilaterally blind was considerably higher when using the US definition of blindness compared to the WHO definition. The number of impaired patients was 60% higher by the US SSA impairment criterion than by the WHO criteria. Visual field testing is very important to achieve correct assessment of visual impairment caused by glaucoma.

## Competing interests

The authors declare that they have no competing interests.

## Authors' contributions

AH initiated the study, participated in the analysis and interpretation of data, has revised the manuscript, checked and approved the initial and revised manuscripts throughout the process. JA collected and analysed the data, drafted the first manuscript versions in collaboration with AH, and checked and approved the initial and revised manuscripts throughout the process. BB has participated in the study design, and in the analysis and interpretation of data, has revised the manuscripts, checked and approved the final manuscripts.

## Pre-publication history

The pre-publication history for this paper can be accessed here:

http://www.biomedcentral.com/1471-2415/11/31/prepub
